# Feasibility and Effectiveness of the Implementation of Anxiety Screening for Adolescents in a Primary Care Setting

**DOI:** 10.7759/cureus.64409

**Published:** 2024-07-12

**Authors:** Mario Soliman, Lidija Petrovic-Dovat, Jeanne M Logan, Benjamin N Fogel

**Affiliations:** 1 Family Medicine, University of Pittsburgh Medical Center Pinnacle, Lititz, USA; 2 Psychiatry and Behavioral Health, Penn State College of Medicine, Hershey, USA; 3 Pediatrics, Penn State College of Medicine, Hershey, USA

**Keywords:** anxiety screening, anxiety disorders, behavioral health, primary care, preventive care, pediatrics

## Abstract

The United States Preventive Services Task Force recommended routine anxiety screening for children and adolescents in 2022. This study describes the feasibility of routine anxiety screening in a primary care practice. It further examines the effects of routine anxiety screening on anxiety diagnoses and provider behavior in a setting in which anxiety screening was implemented five years in advance of the national guidelines. During the first year of implementation, approximately 80% of patients were screened, and 17% of screens were positive. A retrospective chart review of patients with positive screens found that the majority of positive screens led to a new diagnosis of anxiety and that half of newly diagnosed patients were prescribed an intervention. Screening was associated with an increase in diagnoses of anxiety disorders in the studied population from 9.6% to 13.3% (p<0.0001). Following the initial implementation, screening rates continued to rise, with an eventual plateau of >90%. Anxiety screening in the pediatric primary care setting is feasible and sustainable and led to increased provider recognition of anxiety and meaningful clinical action.

## Introduction

Anxiety disorders are among the most common childhood-onset psychiatric conditions, with recent reports estimating an incidence rate of 7.7% in children aged 3-17 and a lifetime prevalence of 31.9% in children aged 13-18 in the United States [1-3]. Mental health disorders in adolescents, including anxiety, became even more prevalent during the COVID-19 pandemic [4,5]. Anxiety disorders in adolescents are associated with educational underachievement and co-occurring psychiatric conditions, as well as functional impairments that can extend into adulthood [6-12]. Despite the high prevalence and significant morbidity, many adolescents with undiagnosed anxiety disorders may wait years before receiving appropriate treatment, resulting in higher morbidity and high social and economic costs [9,13,14].

In light of this, the United States Preventive Services Task Force (USPSTF) recently recommended routinely screening for anxiety in children 8-18 years of age "to identify undiagnosed youth who may benefit from effective treatment for anxiety disorders" [15]. Their recommendation statement acknowledges that more research is needed on the feasibility of using screening tools in the primary care setting [16]. The Screen for Child Anxiety Related Disorders (SCARED) is a 41-item tool with established validity in the primary care setting [17]. We aimed to evaluate the implementation of a short anxiety screening tool, the 9-question Generalized Anxiety subscale of the SCARED (SCARED_GAD_), in a pediatric primary care setting. Because this implementation occurred five years prior to the publication of national guidelines, we are able to report on the effect of the implementation of routine anxiety screening on anxiety diagnoses and provider behavior. Finally, we comment on the sustainability of this implementation.

## Materials and methods

This retrospective chart review study was approved by the Institution Review Board of the Penn State College of Medicine as a non-human subject research. This manuscript uses the appropriate reporting standards for this work, specifically the Standards for Reporting Implementation Science (StaRI) style.

Study population

Participants, selected through convenience sampling, were patients from a single academic primary care pediatric practice in central Pennsylvania that serves approximately 12,500 patients and employs 19 attending physicians, 21 resident physicians, and four nurse practitioners. Patients in the practice are about 80% White, 8% Black, and 10% Hispanic. Approximately two-thirds of patients have private insurance.

Anxiety screening procedure

Beginning April 1, 2017, all patients 11 years of age and older presenting for well-child visits received a printed version of SCARED_GAD_. These forms were included on the reverse side of the Patient Health Questionnaire-9 Modified for Adolescents (PHQ-A) which was this practice's pre-existing standard depression screening tool. The SCARED_GAD_ consists of nine of the phrases (Table 1) from the full SCARED [17]. After the patient filled out the SCARED_GAD_, a nurse would transcribe the responses into the electronic medical record (EMR) system where results are stored in discrete fields and available for provider review. As recommended in the SCARED scoring guide, a score of ≥9 on the Generalized Anxiety subscale indicates a positive screen. Providers were counseled to follow up any positive screen with a clinical interview to further assess anxiety risk and need for intervention.

**Table 1:** Items on the Generalized Anxiety subscale of the Screen for Child Anxiety Related Disorders. Items on the Generalized Anxiety subscale of the Screen for Child Anxiety Related Disorders (SCARED_GAD_). A score of 9 or higher indicates a positive screening result.

Phrase	Scores
I worry about other people liking me.	0=not true. 1=sometimes true. 2=very true.
I am nervous.	
I worry about being as good as other kids.	
I worry about things working out for me.	
I am a worrier.	
People tell me that I worry too much.	
I worry about what is going to happen in the future.	
I worry about how well I do things.	
I worry about things that have already happened.	

Measurements and data collection

The feasibility of implementing anxiety screening in the primary care pediatric setting was measured by tracking screening rates over the first year of implementation. Inclusion criteria were as follows: (1) patients who had a well-child visit between April 1, 2017, and March 31, 2018, and (2) patients aged 11 years or older and under 19 years at the time of the well-child visit. Exclusion criteria included (1) patients who were not in-person in the office, (2) patients who were seen for urgent care, and (3) patients lacking IRB access for verification. The data abstraction tool within the EMR was used to identify the proportion of patients with well-child visits who had a SCARED_GAD_ documented at their visit and to determine the proportion of completed screens that were positive. The proportion of patients who completed an anxiety screener at their well-child visit was tracked monthly using a statistical process control chart (p-chart). Positive screen rates are reported descriptively. 

The effect of screening on provider behavior was analyzed by chart review. A member of the study team completed a retrospective chart review of all patients with positive anxiety screens to identify. The patient's chart was reviewed for (1) recognition (any acknowledgment in the provider documentation that the anxiety screen was positive or a new diagnosis of an anxiety disorder at the visit), (2) diagnosis (a new diagnosis of an anxiety disorder either in the note or in the problem list), and (3) intervention (a new referral to psychiatry, new referral to counseling, and/or new prescription of a medication to treat anxiety). Chart review data was summarized descriptively.

The effect of screening on the diagnosis of anxiety was examined at a population level. The data abstraction tool within the EMR was used to identify the proportion of active (seen for any visit within the prior 36 months) 11- to 18-year-old patients who carried an ICD-10 diagnosis of anxiety before (on March 30, 2017) and one year after (on March 30, 2018) implementation of screening. ICD-10 diagnoses included all F41.X (anxiety disorders), F43.22 and F43.23 (adjustment disorders with anxiety), F40.X (specific phobias), and F93.0 (separation anxiety disorder). A chi-squared test was used to analyze the difference in proportions.

To assess the sustainability of routine anxiety screening beyond the study period, we monitored the ongoing proportion of well-child visits where the SCARED_GAD_ was completed until December 2019. This monitoring involved the use of p-charts, a method specifically designed for tracking process performance over time. P-charts plot the proportion of visits with completed SCARED_GAD_ screenings against time, allowing us to visually assess any shifts or trends in screening adherence. This analytical approach helps identify whether the implementation of anxiety screening remains consistent and sustainable over an extended period, providing insights into the long-term effectiveness and stability of the screening protocol in the pediatric primary care setting.

## Results

A total of 2,275 patients aged 11-18 years old had a well-child visit between April 1, 2017, and March 30, 2018. Approximately 80% (1817/2,275) of patients with well-child visits had an anxiety screen result documented in the EMR on the day of their well-child visit, of which approximately 17% (306/1,817) screened positive. While all age groups had patients with positive screens, the highest proportions were observed in the older age groups (ages 14-18) as shown in Table 2. 

**Table 2:** Percentage of positive screens on the Generalized Anxiety subscale of the Screen for Child Anxiety Related Disorders, categorized by age groups. Percentage of positive screens on the Generalized Anxiety subscale of the Screen for Child Anxiety Related Disorders (SCARED_GAD_), categorized by age groups.

Age	Positive screen	Total screened
11	41 (12%)	352
12	29 (10%)	279
13	40 (16%)	257
14	46 (19%)	246
15	44 (20%)	222
16	50 (24%)	210
17	32 (22%)	147
18	24 (23%)	104
Total	306 (17%)	1817

A retrospective chart review of the 306 patients with positive screens showed that a provider recognized 70% (214) of the positive screens. Primary care providers made a new diagnosis of an anxiety disorder in 82% (176/214) of patients with a recognized positive screen. Of the 176 newly diagnosed patients, 64% (112) received an intervention during their visit. These interventions included 77 referrals to counseling, 15 referrals to psychiatry, and 20 new prescriptions for selective serotonin reuptake inhibitors (SSRIs). 

Population-level analysis of ICD-10 diagnosis data showed that prior to implementing routine anxiety screening, 9.6% (364/3776) of active patients (seen for any visit in the last 36 months) between the ages of 11 and 18 had an ICD-10 diagnosis of anxiety on their problem list. One year after implementing the screening program, 13.3% (520/3898) of active patients 11-18 years of age had an ICD-10 diagnosis of anxiety on their problem list (p<0.0001). 

**Figure 1:**
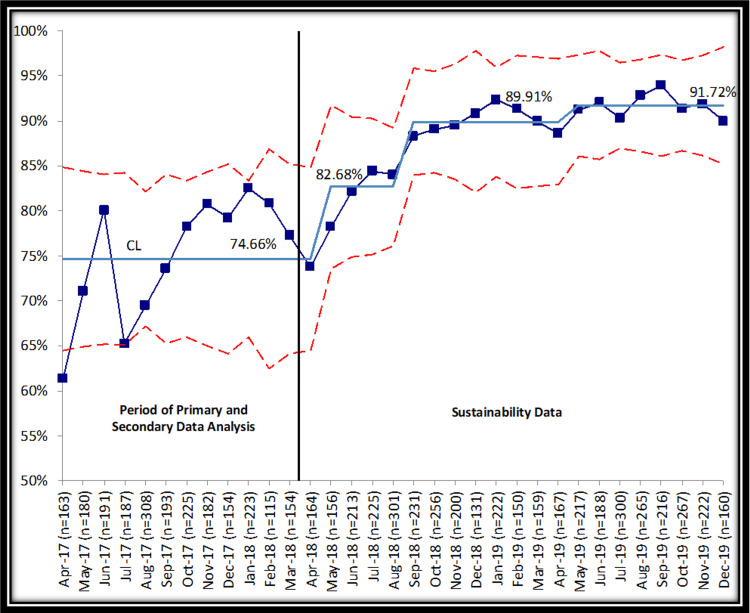
Percent of patients aged 11-18 with a documented SCAREDGAD during their well-child visit. The graph illustrates the percentage of patients aged 11-18 with a documented SCARED_GAD_ during their well-child visit. The vertical line indicates the separation between the study period (April 2017 to March 2018) and the sustainability period (beyond March 2018 to December 2019). "CL" denotes the centerline, while dotted lines indicate the upper and lower control limits.

Sustainability data indicated a continued increase in the documentation of anxiety screening after the study ended in March 2018 with an eventual plateau at >90% (Figure 1).

## Discussion

The high prevalence and morbidity of anxiety disorders in childhood and adolescence and the availability of effective treatments have led the USPSTF to recommend routine anxiety screening in children 8-18 years of age [15,18]. In their recommendation statement, the USPSTF calls for more research on the feasibility of screening in this age group [15,18]. We report on a successful implementation of routine anxiety screening in children 11-18 years of age in the pediatric primary care setting. Screening was not only feasible but was also associated with an intervention (referral to psychiatry, referral to counseling, and/or initiation of a new medication) in 30% (93/306) of patients with positive screens. Routine anxiety screening was also associated with a 39% increase (9.6-13.3%) in the identification of anxiety disorders in our population.

In this retrospective chart review study, we employed a straightforward intervention of adding an anxiety screener to the same piece of paper on which patients received a depression screener. In the 12 months following this intervention, approximately 80% of the 11-18-year-old patients with well-child visits had a documented anxiety screen in their EMR at the time of the well-child visit. Reasons for the lack of documented screen may have included patients not receiving the paper form, patients not completing the paper form, and nurses not entering the results of a completed form into the EMR. While we are not able to comment on the relative effects of these contributing factors, we are encouraged to note that over the 22 months following the end of the study period, and without any specific further intervention, screening rates in this practice setting increased to >90%.

While we found higher rates of positive anxiety screen in the older age groups (ages 14-16), 12% of 11-year-olds and 10% of 12-year-olds completing the screener had an elevated result. This finding supports the USPSTF recommendation to screen for anxiety in this age group. This project predates the USPSTF recommendation to perform anxiety screening beginning at eight years of age, and further research should evaluate the feasibility and effectiveness of screening in this younger age group.

The retrospective chart review points to many important findings that should guide future research. Our finding that providers only recognized and commented on seven out of 10 positive screens may reflect that some clinical interviews following a positive screen for anxiety allay the provider's concerns. It also likely indicates the need for efforts to increase recognition, perhaps through EMR decision supports or alerts. The 17% positive screen rate in our population is in line with the reported 7.7% incidence of anxiety in adolescents [1-3]. Our finding that providers made a new diagnosis of anxiety in over half of patients with a positive screen (and in four out of five recognized positive screens) suggests that screening is identifying clinically relevant cases of anxiety. This is further supported by our population-level analysis, which showed a statistically significant and clinically meaningful increase in the diagnosis of anxiety disorders. Our population prevalence, however, remains well below nationally reported numbers [15,18]. Our finding that 53% of patients newly diagnosed with an anxiety disorder were prescribed an evidence-based intervention supports the assertion by the USPSTF that routine anxiety screening can lead to improved patient outcomes [15,18]. Further qualitative work would be needed to understand the reasons for the lack of intervention in the remaining 47% of newly diagnosed patients, although provider clinical judgment, patient/caregiver preference, and difficulty with access to mental health resources are likely contributors.

Limitations

While this study suggests that implementation of the SCARED_GAD_ is feasible, effective, and sustainable in a primary care setting, there are several important limitations. First, all patients were evaluated at a single primary care site, thus potentially limiting generalizability. While the study population is underrepresented Black and Hispanic when compared with 2018 census data, the proportion of our patients with public insurance closely approximates 2018 census estimates (approximately 1/3). Second, the study was conducted at an academic primary care center with integrated behavioral health, which may not be entirely representative of other primary care centers. Specifically, our providers may have easier access to mental health professionals for formal referrals and informal curbside discussions. Third, this project included patients 11-18 years of age, while the USPSTF now recommends screening beginning at age eight. Finally, the SCARED_GAD_ screens only for generalized anxiety disorder, and, as such, our sample may underreport other anxiety disorders. The Generalized Anxiety subscale of the SCARED is also not widely used as a standalone screen, but it does have accepted scoring thresholds and is similar in length to other short anxiety screeners commonly used in primary care. It's important to consider the next steps that involve improving provider recognition of positive screens, expanding implementation to diverse practice environments to evaluate its applicability, and assessing the effectiveness of alternative screening tools to enhance outcomes. 

## Conclusions

In this study, we report on a feasible and effective implementation of a short anxiety screening tool, the Generalized Anxiety subscale of the Screen for Child Anxiety Related Disorders (SCARED_GAD_), in a pediatric primary care setting. We found that a clinically meaningful proportion of 11- to 18-year-old patients screened positive for generalized anxiety disorder and that providers recognized the majority of these positive screens. We also found that most recognized positive screens led to a new diagnosis of an anxiety disorder and that over half of patients with new diagnoses were prescribed an intervention. While we found a clinically meaningful increase in the identified prevalence of anxiety disorders in our population, our prevalence after implementation still fell short of reported national rates. Finally, we found that the screening program was sustainable, with 90% of patients having documented screens in the post-study period. The next steps should involve efforts aimed at increasing provider recognition of positive screens, expanding this work to varied practice settings to evaluate generalizability, and implementing other available screening tools to evaluate their relative effectiveness.
